# Neutrophil-Derived MMP-8 Drives AMPK-Dependent Matrix Destruction in Human Pulmonary Tuberculosis

**DOI:** 10.1371/journal.ppat.1004917

**Published:** 2015-05-21

**Authors:** Catherine W. M. Ong, Paul T. Elkington, Sara Brilha, Cesar Ugarte-Gil, Maite T. Tome-Esteban, Liku B. Tezera, Przemyslaw J. Pabisiak, Rachel C. Moores, Tarangini Sathyamoorthy, Vimal Patel, Robert H. Gilman, Joanna C. Porter, Jon S. Friedland

**Affiliations:** 1 Infectious Diseases and Immunity, Hammersmith Campus, Imperial College London, London, United Kingdom; 2 Division of Infectious Diseases, Department of Medicine, Yong Loo Lin School of Medicine, National University of Singapore, Singapore; 3 National Institute of Health Research (NIHR) Respiratory Biomedical Research Unit, Faculty of Medicine, University of Southampton, Southampton, United Kingdom; 4 Instituto de Medicina Tropical Alexander Von Humboldt, Universidad Peruana Cayetano Heredia, Lima, Peru; 5 The Heart Hospital, University College London Hospitals, London, United Kingdom; 6 Department of International Health, Johns Hopkins Bloomberg School of Public Health, Baltimore, Maryland, United States of America; 7 Asociación Benéfica Proyectos en Informatica, Salud, Medicina, y Agricultura (PRISMA), Universidad Peruana Cayetano Heredia, Lima, Peru; 8 Centre for Inflammation and Tissue Repair, Department of Medicine, University College London, London, United Kingdom; University of Massachusetts, UNITED STATES

## Abstract

Pulmonary cavities, the hallmark of tuberculosis (TB), are characterized by high mycobacterial load and perpetuate the spread of *M*. *tuberculosis*. The mechanism of matrix destruction resulting in cavitation is not well defined. Neutrophils are emerging as key mediators of TB immunopathology and their influx are associated with poor outcomes. We investigated neutrophil-dependent mechanisms involved in TB-associated matrix destruction using a cellular model, a cohort of 108 patients, and in separate patient lung biopsies. Neutrophil-derived NF-kB-dependent matrix metalloproteinase-8 (MMP-8) secretion was up-regulated in TB and caused matrix destruction both *in vitro* and in respiratory samples of TB patients. Collagen destruction induced by TB infection was abolished by doxycycline, a licensed MMP inhibitor. Neutrophil extracellular traps (NETs) contain MMP-8 and are increased in samples from TB patients. Neutrophils lined the circumference of human pulmonary TB cavities and sputum MMP-8 concentrations reflected TB radiological and clinical disease severity. AMPK, a central regulator of catabolism, drove neutrophil MMP-8 secretion and neutrophils from AMPK-deficient patients secrete lower MMP-8 concentrations. AMPK-expressing neutrophils are present in human TB lung biopsies with phospho-AMPK detected in nuclei. These data demonstrate that neutrophil-derived MMP-8 has a key role in the immunopathology of TB and is a potential target for host-directed therapy in this infectious disease.

## Author Summary

Neutrophil infiltration is characteristic of immune-induced pathology in tuberculosis but mechanisms whereby neutrophils cause tissue destruction are not fully understood. In this study, we show that neutrophils secrete the collagenase MMP-8 in response to direct infection with *Mycobacterium tuberculosis* and via cellular networks. MMP-8 is up-regulated in respiratory samples from TB patients, driving matrix destruction associated with neutrophil activation and reflects disease severity. Neutrophils are present adjacent to the wall of TB cavities in human histology specimens. The metabolic pathway AMP-activated protein kinase (AMPK) regulates neutrophil MMP-8 secretion with data supported by studies in human neutrophils from AMPK-deficient patients. Host-directed therapy against neutrophil MMP-8 may reduce innate-immune mediated tissue damage in TB.

## Introduction

The lung cavity is a hallmark of pulmonary tuberculosis, a globally important disease of man. The cavity has high bacillary burden and is associated with spread of infection. Polymorphonuclear leukocytes or neutrophils are abundant in areas of TB lung cavities [1]. Excessive neutrophil recruitment associates with pathology in animal models [2, 3] and in man [4] but the mechanism of how neutrophils drive pathology in human TB is not defined.

Zinc-containing matrix metalloproteinases (MMPs) have key roles in the inflammatory immunopathology in a wide range of diseases including cancer and arthritis [5, 6]. On the basis of diverse evidence, it has been shown that a matrix-degrading phenotype develops in TB in which MMP activity is relatively unopposed by the specific tissue inhibitors of metalloproteinases (TIMPs) [7]. MMPs are crucial in granuloma formation in the zebrafish model of TB [8] and may drive different stages of lung pathology. Collagenases, a subgroup of the MMPs, are key in TB pathology since collagen is the main structural protein of the lung, the primary site of infection. Patients with pulmonary TB have increased collagenases which correlate significantly with radiological markers of tissue destruction [9, 10]. Neutrophils secrete MMP-8, a potent collagenase, and increased neutrophil-derived MMPs associate with disease severity in CNS-TB [11, 12], implicating neutrophils in the immunopathology of human TB.

The concept of metabolism regulating host immunity is only recently emerging [13]. Adenosine monophosphate-activated protein kinase (AMPK), a serine/threonine kinase is a central regulator of metabolic responses acting as an activator of cellular catabolism [13]. In addition, AMPK is known to have a role in immune responses determining the effector versus memory fate of CD8 T-cells [14]. Inhibition of glucose uptake and AMPK inhibition impedes T cell chemotaxis [15]. Dissecting the mechanism of how metabolism regulates immunity may be key to understanding immunity in chronic infections such as TB.

We hypothesize that neutrophils drive tissue destruction in human pulmonary TB. Animal models of infection such as murine strains which develop pulmonary necrosis and cavities are useful in dissecting areas of the immune response in TB [16–18]. Murine models also demonstrated the critical importance of IFN-γ and TNF-α in the host defence against TB [19–21]. However, there are inherent differences between murine and human neutrophils with divergences in cytokine secretion [22], peptides such as defensins [23], and intracellular signalling pathways [24]. Therefore, in this study, we investigate the role of the neutrophil in MMP-dependent tissue destruction in human pulmonary TB, a disease that affects man as the primary host, and examine the signaling pathways regulating this process.

First, we investigate the effect of neutrophil-derived collagenase MMP-8 in a human cellular model and examine MMP-8 expression and collagenolytic activity in patients. Neutrophils secrete MMP-8 on direct *M*.*tb* infection and in *M*.*tb*-infected monocyte-dependent networks. Neutrophil MMP-8 is expressed in TB patients’ biopsy specimens, with the secretion of MMP-8 dependent on NF-kB. We found in a cohort of 108 TB patients and controls that increased MMP-8 is closely associated with neutrophil markers and correlates with radiological and clinical disease severity. Sputum MMP-8 from TB patients is functionally active, causing matrix destruction, and patients with pulmonary cavities on chest radiographs have higher MMP-8 concentrations in their respiratory secretions. Gelatin degradation in the respiratory samples is raised, but is not dependent on neutrophil gelatinase MMP-9. We demonstrate that AMPK regulates MMP-8 dependent tissue destruction, both at the level of protein secretion and gene expression, using data from a cellular model of infection and by investigating biopsy samples from TB patients and immune responses in AMPK deficient patients. Taken together, our data demonstrate that neutrophils cause tissue destruction in TB by an MMP-8-dependent process, regulated by the pro-catabolic AMPK pathway.

## Results

### Neutrophil MMP-8 is increased in TB and is expressed in neutrophils in patients with pulmonary TB

**Fig 1:**
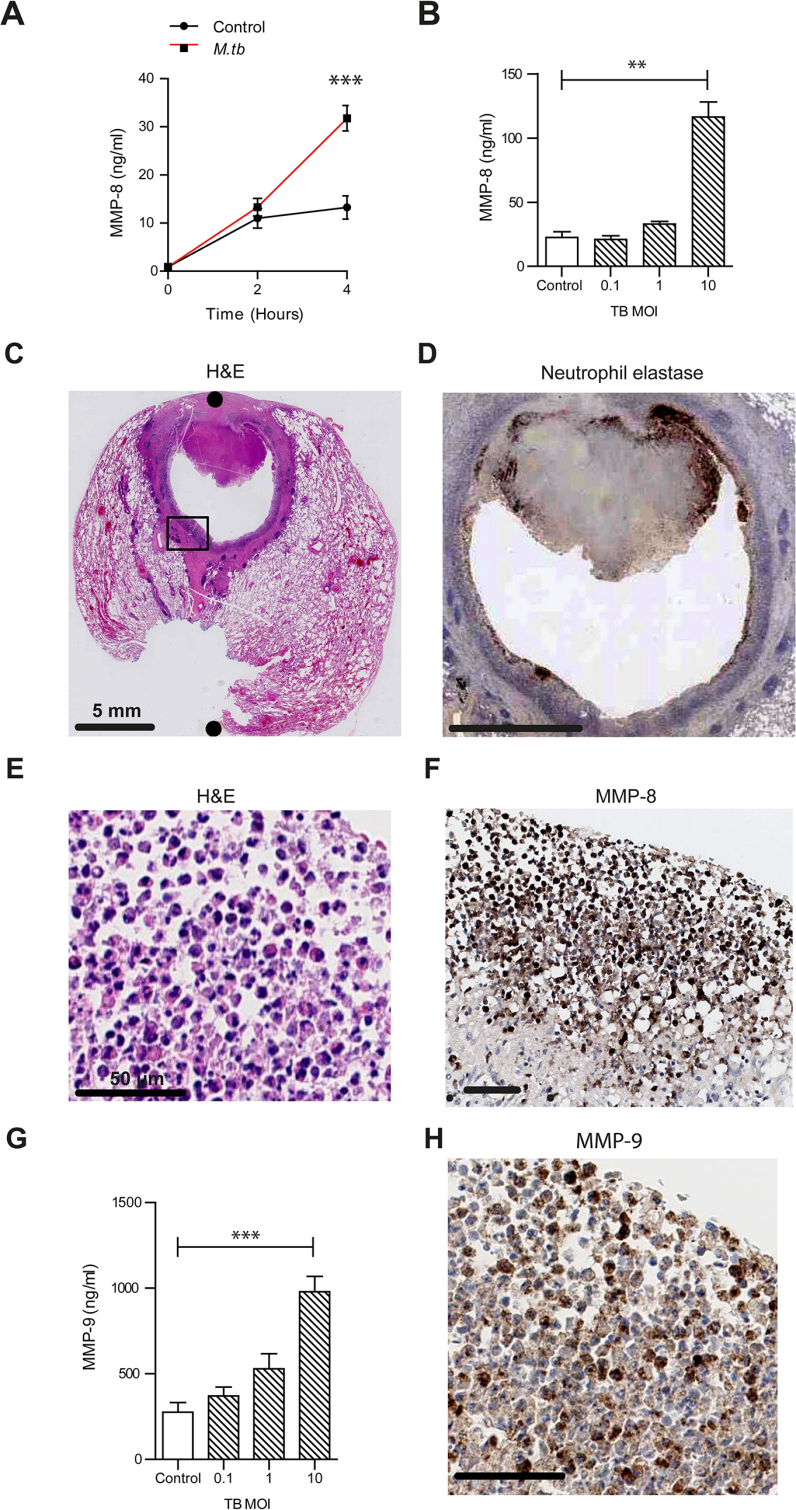
Neutrophil MMP-8 and -9 are upregulated in human TB. (**A**) Neutrophils were infected with *M*.*tb* MOI of 1. MMP-8 secretion was upregulated at 4h. (**B**) Increasing *M*.*tb* MOI caused greater neutrophil MMP-8, analyzed at 4h. Bars represent mean ± s.d. of experiments performed in triplicate and data are representative of a minimum of 2 independent experiments. (**C and D**) Human TB lung biopsy specimens stained with H&E and anti-neutrophil elastase shows neutrophil infiltration around the cavity wall. Both scale bars represent 5 mm. n = 5. (**E and F**) Magnified H&E and MMP-8 stains from Fig 1C inset shows neutrophils immunoreactive for MMP-8 around the cavity wall. Both scale bars represent 50 μm. (**G**) MMP-9 concentrations increase in a dose-dependent manner after *M*.*tb* infection at 4 hours. Bars represent mean ± s.d. of experiments performed in triplicate and data are representative of a minimum of 2 independent experiments. *** *P*<0.001 (**H**) Biopsy proven *M*.*tb* infected human lung specimens were stained for MMP-9 (inset from Fig 1C). Neutrophils were immunoreactive for MMP-9. Scale bar represents 50 μm. Statistical analysis was performed using two-way ANOVA with Bonferroni post-test or One-way ANOVA with Tukey’s post-test. **P<0.01, ***P<0.001.

First, we investigated MMP-8 secretion from primary human neutrophils stimulated by live, virulent *M*.*tb*. Neutrophil MMP-8 secretion increased over time and in a dose-dependent manner in response to higher *M*.*tb* multiplicity of infection (MOI) (Fig 1A and 1B). TIMP-1 and -2 are the MMP inhibitors secreted by neutrophils [25, 26]. TIMP-1 was not secreted in response to stimulation by *M*.*tb* (S1A Fig). TIMP-2 concentrations increased significantly but to a 20-fold lower concentration than MMP-8 (S1B Fig). We demonstrated that neutrophil MMP-8 secretion was blocked by the NF-kB p65 subunit inhibitor Helenalin (IC_50_ 10–50 μM), in a dose-dependent manner and the effect was maximal at 100 μM (S1C Fig, *P*<0.001). The dose-dependent suppression of neutrophil MMP-8 was replicated with additional specific NF-kB inhibitors caffeic acid phenethyl ester (CAPE) [27] (S1D Fig) and SN50 [28] (S1E Fig). Neutrophil viability was greater than 95% for all conditions by FACS staining with Annexin V and live/dead dye (S2 Fig).

To determine the cellular source of MMP-8 in patients with TB, we analyzed lung biopsies from patients who were diagnosed with pulmonary TB. Polymorphonuclear neutrophils were observed along the entire circumference of the inner wall of cavities on H & E staining (Fig 1C). Neutrophil accumulation was confirmed by specific positive staining for neutrophil elastase (Fig 1D). Neutrophils in the same location stained positive for MMP-8 (Fig 1E and 1F). MMP-8 expression was also found in the central area of necrosis of granulomas (S3 Fig), suggesting that MMP-8 may be associated with the process of necrosis.

To determine if other proteases from neutrophils were similarly up-regulated, we analyzed MMP-9 (neutrophil gelatinase) secretion from *M*.*tb* infected neutrophils. *M*.*tb* caused a dose-dependent increase of MMP-9 secretion (Fig 1G). Furthermore, MMP-9 staining of patient lung biopsy specimens also showed presence of MMP-9 in neutrophils (Fig 1H).

### *M*.*tb* infected and CoMTB-stimulated neutrophils degrade matrix

**Fig 2:**
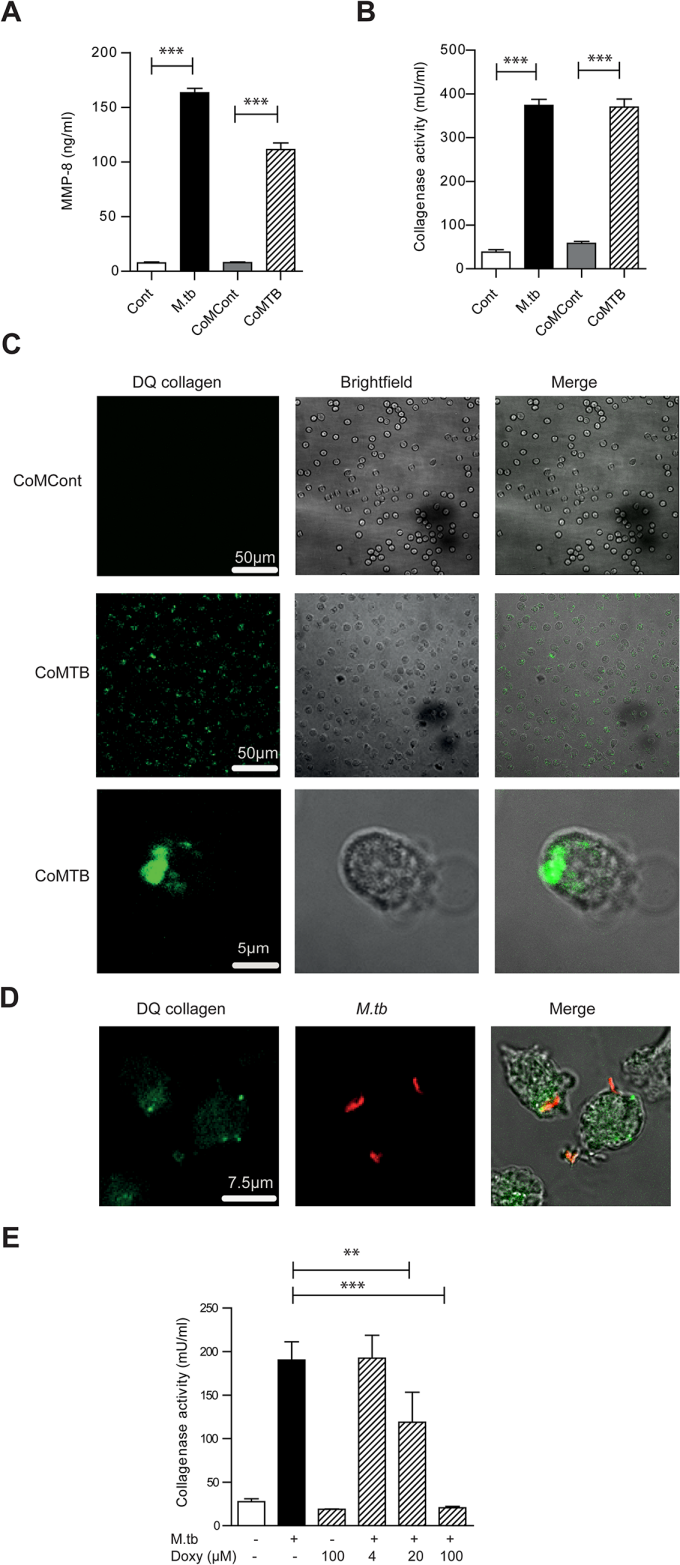
*M*.*tb* and CoMTB-stimulated neutrophils degrade collagen. (**A**) Neutrophils were stimulated with either *M*.*tb* MOI of 10, CoMCont or CoMTB for 4 hours. *M*.*tb* and CoMTB up-regulated MMP-8 secretion analyzed by ELISA. (**B**) Cell-free supernatants from (A) were incubated with Type I DQ collagen. Bars represent mean ± s.d. of experiments performed in triplicate and are representative of a minimum of 2 independent experiments. (**C**) CoMTB caused increased collagen breakdown by neutrophils, resulting in greater fluorescence of DQ collagen. (**D**) Neutrophils were infected with *M*.*tb* MOI 10, fixed and *M*.*tb* stained with *anti-M*.*tb* Ab. Infected cells degraded DQ collagen, increasing fluorescence. (**E**) Cell-free supernatants from neutrophils infected with *M*.*tb* at MOI 10 were added with doxycyline to Type I DQ collagen. Doxycycline suppressed collagenase activity in a dose-responsive manner. Bars represent mean ± s.d of an experiment performed in biological triplicates and represents 2–3 independent experiments. Analysis performed using One-way ANOVA with Tukey’s post-test. ** *P*<0.01, ***P<0.001.

In addition to neutrophils, monocytes are among the early cells to be recruited in *M*.*tb* infection [29] and substantial cross-talk may occur between neutrophils and monocytes [30]. Using conditioned media from monocytes infected by *M*.*tb* (CoMTB) to model intercellular stimulation of neutrophils, we found significant up-regulation of MMP-8 secretion similar to *M*.*tb* infection (Fig 2A). We assessed the functional consequences of MMP-8 activity on degradation of Type I collagen, the main extracellular matrix fibril providing structural support in human lung parenchyma [31]. Both *M*.*tb*-infected and CoMTB-stimulated neutrophils degrade DQ collagen as assessed by a quantitative fluorescence assay (Fig 2B). Confocal microscopy demonstrated collagen degradation at the neutrophil-collagen interface both in *M*.*tb*-infected and CoMTB-stimulated neutrophils (Fig 2C and 2D). There was dose-dependent inhibition of collagenase activity to baseline when neutrophil supernatants were treated with doxycycline, an MMP inhibitor licensed in the USA for use in periodontal disease [32]. The effect was maximal after treatment with 100 μM doxycyline (Fig 2E, *P<0*.*001*).

### Neutrophil MMP-8 is found on NETs

**Fig 3:**
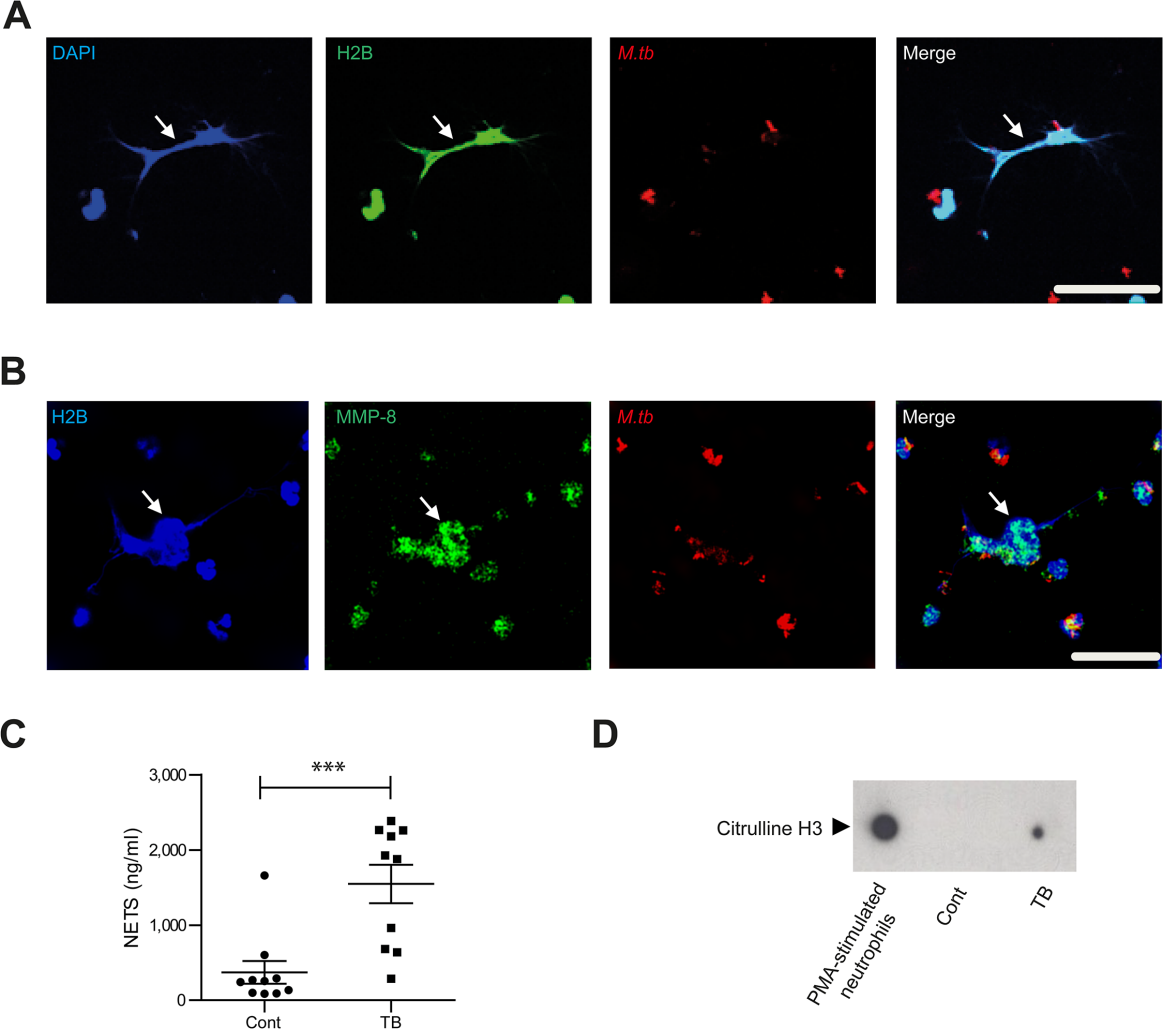
Neurophil MMP-8 associates with NETs. (**A** and **B**) Neutrophils were infected with *M*.*tb* MOI of 10 for 4 hours and NETs stained with DAPI (blue), anti-histone 2B (H2B, green) or anti-MMP-8 (green), while *M*.*tb* was stained with anti-*M*.*tb* Ab (red). *Mtb* induces NET formation which do not adhere to the shape of the neutrophil nuclei (White arrows). Scale bars represent 25 μm. (**C**) Induced sputum NETs were greater in patients with TB than healthy controls (*n* = 10 both groups analyzed by Student’s t-test). (**D**) NETs marker citrulline H3 is present in induced sputum of TB patients but not in healthy controls. Blot representative of *n* = 2 both groups.

Next we showed that neutrophils generate NETs when stimulated with *M*.*tb in vitro* (S4A Fig), and NETs were digested by DNAse (S4B and S4C Fig). Neutrophil extracellular traps (NETs) are scaffolds containing DNA, histones and antimicrobial granule proteins. We demonstrated for the first time that MMP-8 co-localizes with NETs (Fig 3A and 3B). Next, we evaluated NETs in induced sputum from TB patients and healthy controls from a clinical study [33] (S1 Table). Sputum from TB patients had increased NET concentrations of 1548 mg/ml (± standard error 256 mg/ml) compared to controls at 372 mg/ml (± S.E. 150mg/ml) (Fig 3C, *P*<0.001). Citrulline H3, an established marker of NETs [34, 35] was detected in induced sputum of TB patients and not in healthy controls (Fig 3D). This was not due to dead or dying cells since these neutrophils do not contain citrulline H3 (S4D Fig).

### MMP-8 in induced sputum of TB patients is closely associated with neutrophil markers and drives matrix degradation

**Fig 4:**
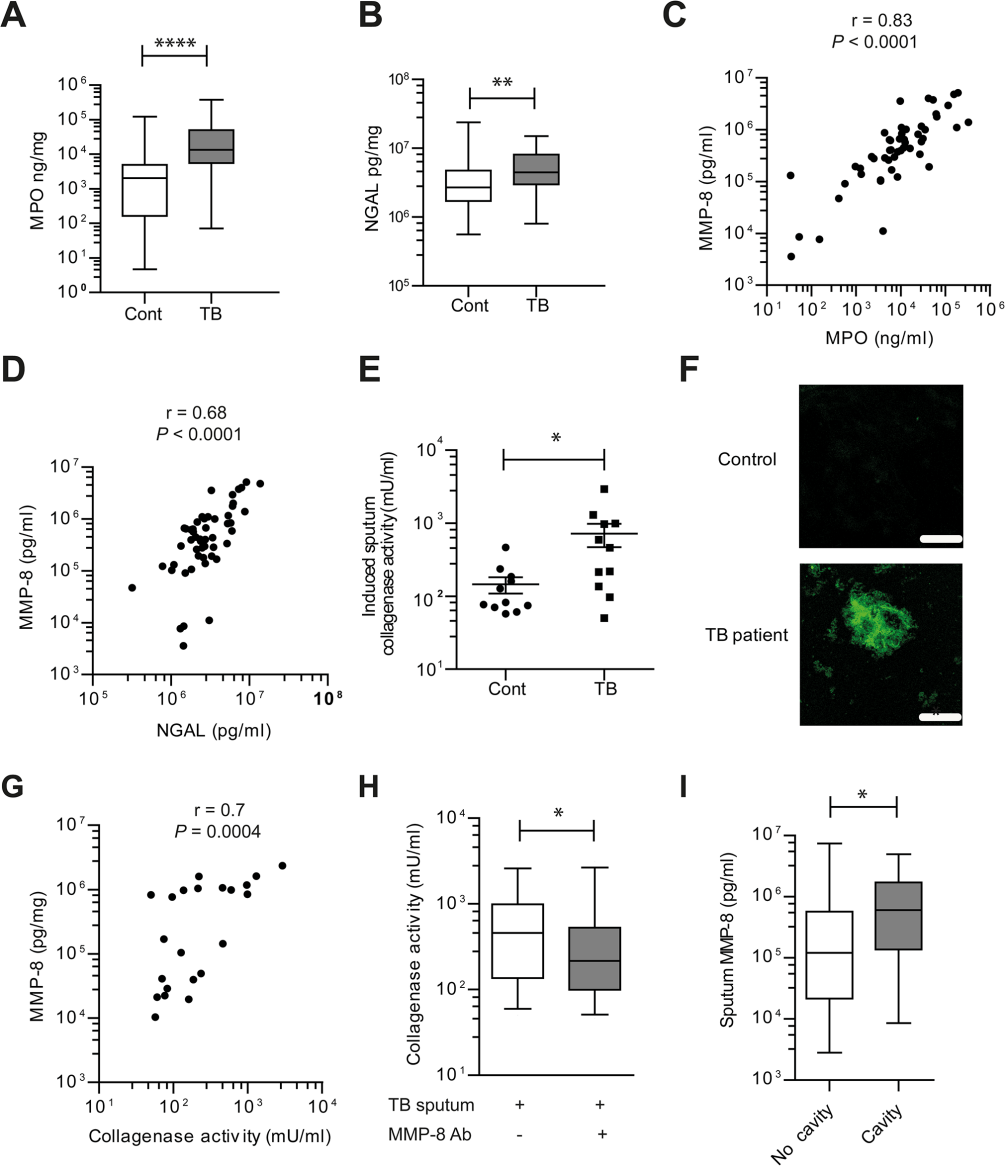
Induced sputum samples of pulmonary TB patients have increased collagenase activity due to neutrophil-derived MMP-8. (**A and B**) Induced sputum MPO and NGAL was analyzed by ELISA from *n* = 51 TB patients and *n* = 57 healthy controls. MPO and NGAL were increased in patients with pulmonary TB. (**C and D**) Induced sputum MMP-8 closely correlated with both MPO and NGAL in TB patients, performed using Spearman’s correlation coefficient. (**E**) Induced sputum collagenase activity is increased in TB patients. (*n* = 11 each group). Subsets analyzed were representative of the whole cohort analyzed by Mann-Whitney test. (**F**) Confocal microscopy shows increased DQ collagen degradation in induced sputum of TB patient relative to control. Image is representative of *n* = 3 each group. Scale bars represent 50μm. (**G**) Induced sputum MMP-8 and collagenase activity correlate, analyzed by Spearman’s correlation coefficient (*n* = 22). (**H**) MMP-8 neutralization suppresses induced sputum collagenase activity from TB patients. MMP-8 neutralizing antibody was added to activated induced sputum with Type I DQ collagen (*n* = 11). Box and whiskers represent 10–90^th^ percentile with comparison using Wilcoxon-Sign rank test. (**I**) Induced sputum MMP-8 were higher in patients with pulmonary cavities than those without. * *P*< 0.05, **P<0.01, ****P<0.0001.

MMP-8 is substantially elevated in the induced sputum of TB patients compared to other MMPs [33]. To determine if MMP-8 was neutrophil derived, we analyzed two established markers of neutrophil activation, myeloperoxidase (MPO) and neutrophil gelatinase associated lipocalin (NGAL) [36, 37] in induced sputum. In a cohort of 51 TB patients and 57 healthy controls randomly selected from our previously reported study of 137 patients [33], MPO and NGAL concentrations were increased in induced sputum of TB patients compared to controls (Fig 4A and 4B). Both sputum MPO and NGAL concentrations correlated strongly with MMP-8 (r = 0.83, *P*<0.0001 and r = 0.68, P<0.0001 respectively) (Fig 4C and 4D), indicating that MMP-8 in induced sputum of TB patients is likely to be principally derived from neutrophils.

Next, we demonstrated that induced sputum from TB patients had increased collagenase activity compared to healthy controls using the DQ collagen degradation assay (Fig 4E, *P* = 0.02), confirmed on confocal microscopy (Fig 4F). Sputum MMP-8 concentrations strongly correlated with collagenase activity (r = 0.7, *P* = 0.0004) (Fig 4G) and MMP-8 neutralization decreased collagenase activity in respiratory secretions of TB patients (*P* = 0.01) (Fig 4H). When the cohort was stratified according to the presence or absence of lung cavities, patients with pulmonary cavitation secreted a median of 5-fold higher MMP-8 concentration than those without cavities. (*P*<0.028, Fig 4I). In addition, MMP-8 sputum concentrations positively correlated with the TB score (r = 0.56 for n = 108; *P*<0.0001), a clinical marker of disease severity. The other major neutrophil-derived MMP, MMP-9, had a much weaker although statistically significant correlation with TB score (r = 0.3453 for n = 108; *P* = 0.003). Analyzing CXR consolidation score as a radiological marker of tissue destruction demonstrated a similar strong MMP-8 correlation (r = 0.52 for n = 74; *P*<0.0001) and a weaker MMP-9 correlation with pathology (r = 0.31 for n = 74; *P* = 0.0077).

To determine if MMP-9 contributes to matrix destruction in TB patients, we assessed the gelatinase activity of the respiratory secretions. Induced sputum from TB patients showed an increased gelatinase activity (*P*<0.0001; S5A Fig). However, MMP-9 neutralization with an inhibitory antibody at 10 μg/ml which completely suppresses gelatinase activity from MMP-9 [38], did not decrease gelatinase activity in the respiratory secretions (S5B Fig).

### AMP-activated protein kinase regulates neutrophil MMP-8 secretion in TB in vitro

**Fig 5:**
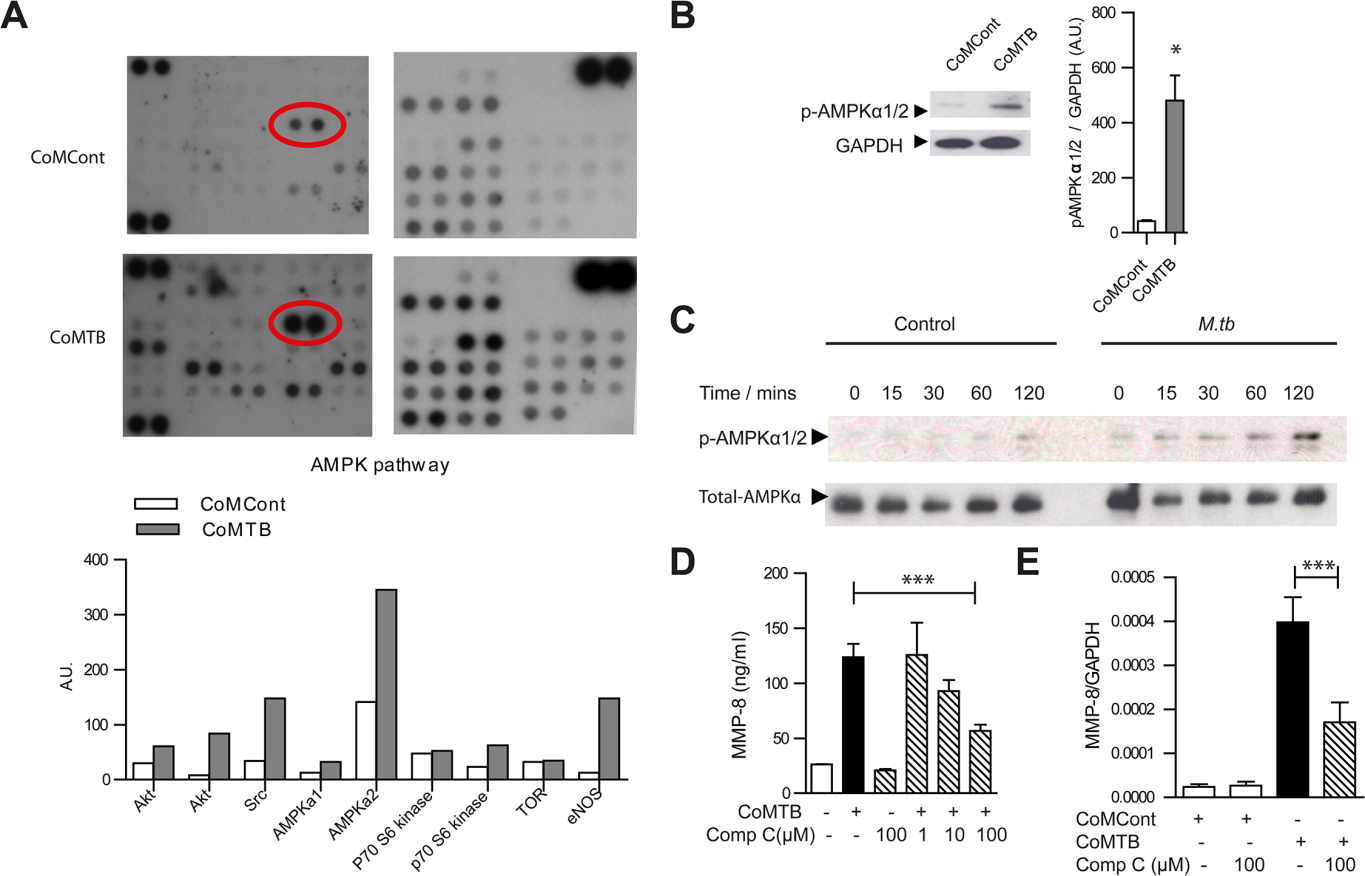
AMPK regulates neutrophil MMP-8 secretion in TB *in vitro*. (**A**) Human phosphokinase array. Neutrophils were stimulated with CoMCont or CoMTB. Representative blot from *n* = 4 healthy donors. Red circle highlights increased AMPKα2 phosphorylation in CoMTB-stimulated cells, with densitometric analysis of components of AMPK pathway below. (**B**) CoMTB stimulation phosphorylates AMPKα1/2 analyzed by western blotting and gel densitometry. Neutrophils were stimulated for 30 minutes. Bars represent mean ± s.e.m from *n* = 3 donors. (**C**) Neutrophils were infected with *M*.*tb* MOI of 10 and cell lysates immunoblotted for phospho-AMPKα (T172) at defined time points. *M*.*tb* caused maximal phosphorylation at 120 mins. (**D**) Compound C (Comp C) pre-incubation for 30 minutes before CoMTB stimulation suppresses neutrophil MMP-8 secretion at 4 hours. (**E**) Compound C (Comp C) was pre-incubated for 30 minutes before stimulation with CoMCont or CoMTB for 24 hours with MMP-8 gene expression analyzed by real-time PCR normalized to GAPDH. Bars represent mean ± s.d. of an experiment performed in biological triplicates on at least 2 occasions. *P<0.05, ***P<0.001. Analysis was performed using one-way ANOVA with Tukey’s post-test.

To investigate the key regulatory pathways of neutrophil MMP-8 secretion, we performed a screening human phosphokinase array and observed that the AMP-activated protein kinase (AMPK) pathway was consistently activated in *M*.*tb*-infected neutrophils, especially AMPKα2 (T172) (Fig 5A). This activation was confirmed by immunoblotting (Fig 5B). Components of the MAP-kinase, STAT pathways, p53 and Src family of kinases were also activated consistent with previous data [39–41] (S6A, S6B and S6C Fig). AMPK is considered a master regulator of cellular energy homeostasis, existing as a heterotrimeric complex comprising catalytic α-subunits and regulatory β- and γ-subunits [13]. Its activation sets off a cascade of catabolic pathways including glycolysis and ketogenesis which can lead to the wasting which is characteristic in TB patients. We demonstrated that AMPKα was activated by phosphorylation in neutrophils directly infected with *M*.*tb* (Fig 5C).

The specific AMPK inhibitor Compound C (Comp C) blocked neutrophil MMP-8 secretion in a dose-dependent manner towards baseline levels (Fig 5D) and also suppressed gene expression of neutrophil MMP-8 (Fig 5E), confirming AMPK is functionally active in regulating neutrophil MMP-8 secretion. Since the AMPK pathway may be downstream of the Akt/PI3-kinase pathway [42, 43] and the Akt/PI3-kinase pathway may drive tissue destruction in TB [44], we investigated whether this path regulates human neutrophil MMP-8 secretion. Neutrophil Akt was phosphorylated in response to CoMTB (S7A Fig) but MMP-8 secretion was not suppressed by either the Akt-inhibitor (Akt-i) or the broad-spectrum PI3-kinase inhibitor LY 294002 (S7B and S7C Fig). We also examined whether the mTOR/p70S6 kinase regulated neutrophil MMP-8 secretion as this is downstream of AMPK [45]. p70S6 kinase was phosphorylated in neutrophils stimulated by CoMTB (S7D Fig) but the mTOR inhibitor rapamycin did not inhibit neutrophil MMP-8 secretion (S7E Fig), indicating that neutrophil MMP-8 secretion is independent of this pathway.

### AMPK regulates neutrophil MMP-8 secretion in TB in patients

**Fig 6:**
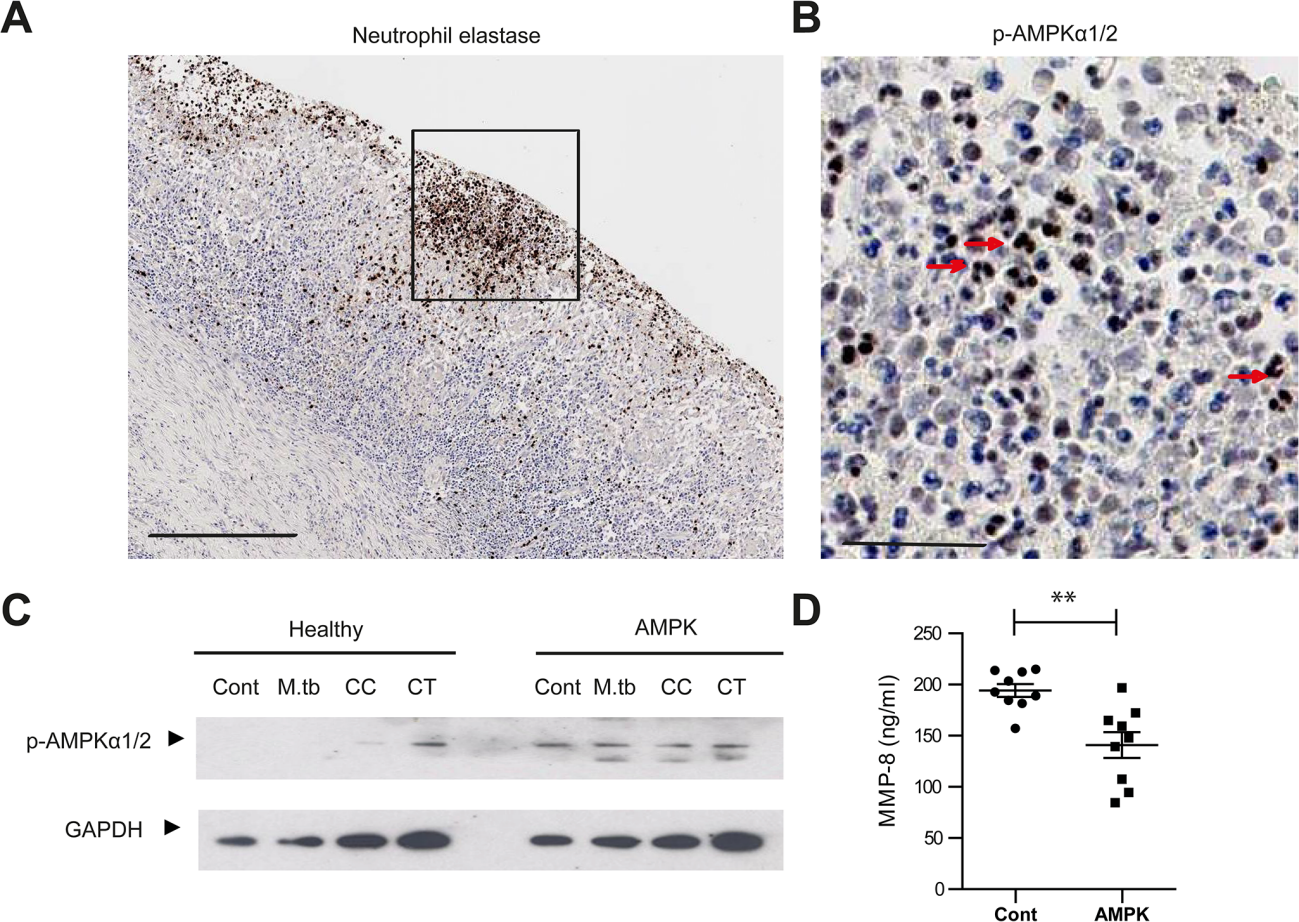
AMPK regulates neutrophil MMP-8 secretion in patients. (**A**) Neutrophils are present in human TB lung cavity wall and stain positive for neutrophil elastase. Scale bar 250μm. (**B**) Neutrophils express phospho-AMPKα (T172) in the nuclei (red arrows) (inset of A). Scale bar 50μm. (**C**) Neutrophils from healthy donors and patients with AMPK deficiency were stimulated with *M*.*tb* MOI of 10, CoMCont or CoMTB for 30 minutes. In patients, AMPK is constitutively phosphorylated, resulting in a functional deficiency. Blot representative of 6 healthy controls and AMPK patients. (**D**) Neutrophils from healthy donors and patients with AMPK deficiency were stimulated with CoMTB. *n* = 3 both groups. Neutrophils derived from AMPK patients secreted less MMP-8 when stimulated than cells from healthy donors. Experiments were performed in biological triplicates and each point represents a sample. Analysis was performed using one-way ANOVA or two-tailed t-test. ** *P*<0.01, ***P<0.001.

Finally, we studied AMPK *in vivo*. In human TB lung specimens, AMPKα was phosphorylated within the nuclei of neutrophils in TB cavities (Fig 6A and 6B), indicating a state of energy depletion [46]. AMPK regulation of neutrophil MMP-8 was further investigated using neutrophils from a group of patients with defects in AMPK activation. Their clinical phenotype is typically similar to those found in glycogen storage disorders [47]. Genotypically, these patients have an AMPKγ2 mutation [48]. AMPKα mutations have not been described in man. In these patients, AMPKα was phosphorylated in both unstimulated and stimulated neutrophils but not in healthy donors, demonstrating increased basal phosphorylation of AMPKα (Fig 6C). Such increased basal AMPK activity reduces the sensitivity of the protein kinase to AMP, resulting in functional AMPK deficiency [47]. MMP-8 secretion from AMPK-deficient neutrophils stimulated by CoMTB was significantly less than MMP-8 secreted by AMPK-replete neutrophils (Fig 6D, *P*<0.01), implicating AMPK in the regulation of neutrophil MMP-8 secretion in man.

## Discussion

Neutrophils are emerging as key mediators of TB-associated inflammation. They drive the unique TB transcript signatures in man [39] and predominate in respiratory secretions of patients with pulmonary TB [1, 49]. We found sputum from TB patients had increased MMP-8 concentrations, neutrophil myeloperoxidase (MPO) and neutrophil gelatinase associated lipocalin (NGAL) compared to controls. MMP-8 was strongly associated with markers of neutrophil activation, MPO and NGAL, indicating that sputum MMP-8 is likely to be neutrophil-derived. Neutrophils expressing MMP-8 were found in the inner walls of tuberculous cavities and may further erode the lung matrix, extending previous findings that neutrophils are the predominant phagocytic cells in the respiratory secretions of TB patients [1]. We also found higher MMP-8 in TB patients with cavities on their chest radiographs than those without cavitation. These findings are consistent with and extend our previous data in smaller groups of patients that demonstrated a trend to increased MMP-8 compared to patients with respiratory symptoms [9, 10]. Furthermore, MMP-8 is significantly elevated in plasma samples of patients with TB compared to respiratory symptomatics [50]. These observations underscore the importance of collagenases, such as MMP-8 and MMP-1, which are the only enzymes capable of degrading the collagen triple helix at neutral pH. The consistent elevation of MMP-8 across the different patient cohorts implicates neutrophils as key players in tissue destruction in TB.

In animal models of TB, neutrophil influx is associated with poorer outcomes with higher bacterial burden, earlier mortality and tissue inflammation [51–53]. However, the mechanisms linking neutrophil excess and poor outcomes are unclear. In our human study, *M*.*tb* drove neutrophil MMP-8 secretion, causing destruction of collagen, the main structural protein in human lung, both *in vitro* and in TB patients. We showed neutrophil MMP-8 closely correlated with sputum collagenase activity as well as clinical CXR score and TB severity score, implicating neutrophils in driving pathology in man by their collagenolytic activity. MMP-8 was also associated with NETs in *M*.*tb* infection and NET components such as citrulline H3, which were not present in dead neutrophils, were increased in the respiratory secretions of TB patients. This may further contribute to immunopathology since NETs are recognized to induce cell death [54, 55]. It is likely that different MMPs predominate in different stages of disease in TB immunopathology. There is good evidence that neutrophils are present not only during the acute phases of TB infection with macrophages, but are also a dominant cell type at the site of established infection of the cavity together with lymphocytes [1, 51, 52]. Such neutrophils contain high concentration of pre-synthesized MMP-8 [56], and so can drive the later stages of TB which leads to lung cavitation, morbidity and death

MMP-8 inhibition may be a target to abrogate excessive host tissue destruction. MMP-8 is a critical mediator of lung parenchymal damage other lung diseases, such as COPD [57] and ventilator-induced lung injury, and MMP-8 inhibition improves outcomes in a murine model of lung injury [58]. We showed higher MMP-8 in the respiratory secretions of patients with cavities than those without and MMP-8 neutralization decreased the matrix destruction in the sputum of TB patients. Although neutrophil gelatinase MMP-9 is secreted in TB and expressed in TB patients, neutralizing MMP-9 did not reduce gelatinase activity in TB patients. Neutrophil MMP-8 secretion in TB was inhibited by NF-kB inhibitors helenalin, CAPE and SN50 without altering neutrophil viability. Furthermore, doxycycline reduced neutrophil collagenase activity to baseline and MMP-8 neutralizing antibody decreased collagen destruction *ex vivo* in TB patients. Such immunomodulatory agents have potential to reduce tissue destruction in TB.

For the first time in TB, we have shown that there is interaction between the metabolic AMPK signaling pathway in the regulation of neutrophil MMP-8 secretion and innate-immune mediated tissue destruction. AMPK activates catabolic pathways such as fatty acid oxidation and glycolysis to generate ATP, while switching off energy-consuming processes including protein and fatty-acid biosynthesis and cell-cycle progression. Two studies have shown that the development of lung injury in murine models is dependent on the pro-catabolic AMPK pathway [59, 60] with AMPK activation decreasing lung injury. This contrasted with our findings where AMPK inhibition decreased neutrophil MMP-8 secretion, and maybe due to AMPK having divergent effects on different cells.

The AMPK pathway was up-regulated in our cellular model and drives neutrophil MMP-8 secretion and gene expression, which was inhibited by the AMPK inhibitor Compound C. This finding was repeated in a small cohort of extremely rare patients with functional AMPK deficiency from a γ_2_-subunit mutation, where *M*.*tb*-driven neutrophil MMP-8 was decreased compared to healthy volunteers. While this does not definitively prove that AMPK regulates MMP-8 secretion as metabolic differences in neutrophils may cause divergent secretion, it implicates AMPK in driving neutrophil-mediated pathology by MMPs in TB. This finding is in keeping with a recent study where AMPKα2 deficient mice had decreased MMP-2 and were found to be resistant to developing abdominal aneurysms, a process which was demonstrated to be MMP-dependent [61]. Recent data demonstrate that AMPK has the ability to shuttle through the nucleus and contains both cytosolic components and nuclear components [46] with the ability to control transcription [62]. We showed AMPKα was phosphorylated in *M*.*tb-*infected neutrophils and adjacent to human TB lung cavities, with phospho-AMPKα located in the nuclei, signifying a state of energy depletion [46].

Together, our data from our human cellular model and in patients demonstrate that neutrophils drive MMP-8-dependent tissue destruction in TB, providing an insight as to how excessive neutrophil infiltration exerts a detrimental effect on the host. This process is controlled by the metabolic regulator AMPK as demonstrated *in vitro* and in AMPK deficient patients. This study highlights a previously unappreciated connection between metabolic paths that directly interact with innate immune responses causing immunopathology in human TB. Interventions specifically targeting the intersection of metabolic and innate immune responses to decrease tissue destruction may improve outcomes in TB and other inflammatory disorders.

## Materials and Methods

### Reagents and antibodies

Mouse anti-human beta-actin, rapamycin and doxycycline hyclate were from Sigma. Helenalin, SN50 and Compound C were from Merck Biochemicals. Caffeic acid phenethyl ester (CAPE) was from Tocris (R&D Biosystems). Mouse anti-human MMP-8, mouse anti-human MMP-9, rabbit anti-human GAPDH, rabbit anti-human histone 2B, rabbit anti-human phospho-p70S6k (T229), rabbit anti-Mycobacterium tuberculosis, rabbit anti-human neutrophil elastase, rabbit anti-human phospho-AMPK alpha 1 and 2 (T172), sheep anti-human histone 2B, rabbit anti-human histone H3 (citrulline 2 + 8 + 17), donkey anti-Sheep IgG DyLight 488, goat anti-mouse DyLight 549, goat anti-rabbit IgG Cy5 were from Abcam. Rabbit anti-human phospho-Akt, total-Akt, phospho-AMPKα1/2 (T172), total AMPKα, and goat anti-rabbit HRP linked were from Cell Signalling Technology. Goat anti-mouse IgG (H+L) was from Jackson Immunoresearch laboratories. Goat anti-human MMP-8 was from R&D Biosystems and mouse anti-human MMP-9 was from Millipore. Rabbit anti-human MMP-8 was from Novus Biologicals. Mouse anti-human neutrophil elastase was from Dako. Mouse anti-human MMP-9 was from Millipore.

### Recruitment of patients and controls

#### Ethics statement

For recruitment of TB patients and controls, the Institutional Review Board from Universidad Peruana Cayetano Heredia and Direccion de Salud Lima Este (Lima, Peru) approved this study and written informed consent was obtained from all participants. For AMPK patients, the study received the Institutional Review Board approval from Central London Research Ethics Committee and written informed consent was obtained from all patients. For extraction of primary human neutrophils, ethical approval for obtaining healthy human volunteer blood was provided by the Outer West London Research Ethics Committee and written informed consent was obtained from individuals. Ethical consent for the study of anonymized paraffin-embedded sections from histopathology was obtained from the Hammersmith Hospitals Research Ethics Committee in accordance with The Human Tissue Act 2004 and written informed consent was obtained from subjects who donated their biopsy specimens.

#### TB patients and controls

A subset of 51 TB and 57 control patients were analysed from the original cohort of 137[33]. In brief, TB patients were recruited prior to starting TB therapy and were microbiologically positive, aged over 18 years, had no prior history of TB or TB treatment and were HIV negative. Healthy controls aged over 18 years had no symptoms associated with TB, a normal chest radiograph and negative sputum TB culture. Induced sputum samples of at least 3 mls was obtained from TB patients and healthy controls and were sterile filtered using a 0.2μm Durapore membrane (Millipore). Total protein concentrations were measured using Bradford assay (Bio-Rad). Disease severity was assessed using an established clinical TB Score [63]. This is a clinical score that evaluated the following: cough, hemoptysis, dyspnea, chest pain, night sweats, conjunctival pallor, tachycardia, axillary temperature above 37°C, body mass index and middle upper arm circumference (MUAC) with a total possible score of 13. Chest radiographs were scored for extent of pulmonary consolidation with Image J 1.43U (NIH, USA) using the formula: (Area of TB consolidation/Total lung area) x (Mean absorbance of TB consolidation/Mean lung absorbance) x 100% as before [33].

#### AMPK patients and controls

The cardiology specialty consultation service of The Heart Hospital, University College London identified patients with cardiomyopathy, who were confirmed on genotyping to have an AMPKγ2 mutation. Neutrophil isolation was performed concurrently from each patient and from a healthy control with cells stimulated concurrently.

### *M*.*tb* culture

*M*. *tuberculosis* H37Rv was cultured in supplemented Middlebrook 7H9 medium (BD Biosciences). For infection experiments, mycobacteria were used at mid-logarithmic growth at an optical density of 0.60 (Biowave cell density meter; WPA).

### Cell culture and stimulation

Whole blood were drawn in preservative-free heparin and mixed with equal volumes of 3% dextran saline to remove erythrocytes. Neutrophils were isolated from the resulting cell suspension using Ficoll-Paque density centrifugation and three rounds of hypotonic lysis. Neutrophil purity was over 95% by FACS and viability >99% by trypan blue assay. In some experiments, neutrophils were pre-incubated with specific inhibitors/agents as indicated for 30 minutes unless otherwise stated. In all experiments involving live *M*. *tuberculosis* H37Rv, tissue culture medium was sterile filtered through 0.2 -μm Anopore membranes (Millipore) before removing from the CL3 laboratory. All experiments were performed using 4 hour incubations unless otherwise stated.

Primary human blood monocytes were prepared from donor leukocyte cones from healthy donors (National Blood Transfusion Service, UK). After density gradient centrifugation (Ficoll Paque) followed by adhesion purification, monocyte purity was over 95% by FACS analysis. Monocytes were infected with *M*.*tuberculosis* at a multiplicity of infection (MOI) of 1. After incubation at 37°C for 24 h, conditioned medium was harvested and was termed CoMTB. Media from uninfected monocytes was termed CoMCont.

### ELISAs for TIMP-1/2, MPO and NGAL

TIMP-1 and 2 concentrations were measured using the Duoset ELISA Development System (R&D Systems) and detected a minimum of 31.2 pg/ml for both. The human MPO Quantikine ELISA kit (R&D Systems) was performed according to manufacturer’s instructions and the lower limit for MPO detection was 0.1 ng/ml. NGAL was measured using the human NGAL ELISA kit (Bioporto Diagnostics) which had minimum detection limit of 1.6 pg/ml.

### Luminex array

MMP-8 and -9 concentrations were analyzed by Fluorokine multianalyte profiling kit according to the manufacturer’s protocol (R&D Systems) on the Luminex platform (Bio-Rad). The minimum level of detection for MMP-8 and -9 was 110 pg/ml and 65 pg/ml respectively. Cytokine concentrations were analyzed using a human 30-plex panel (Invitrogen).

### Human phosphokinase array

The Proteome Profiler Human Phospho-kinase array kit (R&D Systems) which detects 45 phosphorylated proteins was performed according to the manufacturer’s protocol and developed with the ECL system (Amersham Biosciences). Thirty minutes after neutrophils were stimulated with CoMTB, the cells were pelleted and lysed in lysis buffer. Equal amounts of total protein were loaded on to each array. Densitometric analysis of arrays was performed using Scion Image version Beta.4.0.2.

### DQ collagen and gelatin degradation assays

Type I collagen and gelatin degradation was assessed using the EnzChek Gelatinase/Collagenase Assay kits (Molecular Probes). Samples were activated with 2 mM of 4-amino-phenyl mercuric acetate (APMA) for 1 hour at 37°C. 80μL of reaction buffer or inhibitor (doxycyline hyclate, Goat anti-human MMP-8 or Mouse anti-human MMP-9) were added with 20μL of either DQ collagen or gelatin (Invitrogen) at a final concentration of 25μg/ml. Activated samples were subsequently added, and activity detected at specified times using a fluorometer (FLUOstar Galaxy).

### Isolation and quantification of neutrophil extracellular traps (NETs)

Human neutrophils were infected with *M*.*tb* at an MOI of 10 and 20 nM PMA was used as a positive control. 5 U/ml of micrococcal nuclease (Fermentas) was added in each well for 10 minutes at 37°C, after which EDTA was used to halt the reaction. Supernatants were collected, sterile filtered and stored at 4°C. NETs were quantified using QuantiT PicoGreen (Invitrogen) according to manufacturer’s instructions.

### Immunoblotting

Pelleted neutrophils infected with *M*.*tb* or stimulated with CoMTB were mixed with SDS lysis buffer. Samples were run on the NuPAGE 4–12% Bis-Tris gels with SDS Running buffer (Invitrogen). Protein was transferred onto a nitrocellulose membrane (GE Healthcare). Primary antibody was diluted in 5% BSA/0.1% Tween and incubated overnight at 4°C with agitation. Secondary antibody was added diluted in blocking buffer. Luminescence was demonstrated with ECL Substrate Reagent (Amersham Science) according to manufacturer’s instructions and exposing the membrane to Hyperfilm ECL. Densitometric analysis was performed using Image J 1.43U (NIH, USA).

### Real-time PCR

Total RNA was extracted from 2 x 10 ^6^ neutrophils using the RNeasy Mini Kit (Qiagen). Quantitative real-time RT-PCR was performed using the OneStep RT- PCR master mix (Qiagen) according to the manufacturer’s instruction on a Stratagene Mx3000P platform using 5–10 μg per sample. MMP-8 primer and probe mixes were obtained from Applied Biosystems. GAPDH (Forward primer 5’- CGCTTCGCTCTCTGCTCCT-3’, reverse primer 5’- CGACCAAATCCGTTGACTCC-3’, probe 5’-HEX-CGTCGCCAGCCGAGCCACAT-TAMRA-3’) was analyzed in parallel. To accurately determine the quantitative change in RNA, standard curves were prepared from plasmids subjected to real-time PCR as above. MMP-8 data were normalized to GAPDH detected in the same sample.

### Flow cytometry

Cell viability was assessed by staining neutrophils with Annexin V-FITC apoptosis detection kit (eBioscience, Affymetrix, California, USA) and live/dead fixable dead cell stain kit (Invitrogen). Neutrophils were stimulated with 200 ng/ml staurosporine to induce apoptosis and this was used as a positive control for all experiments. Annexin V was detected on the FL-1 channel and live/dead dye on FL-3. A total of 50,000 events were gated and analysed on BD FACSCalibur flow cytometer using CellQuest. Data was analysed using FlowJo 7.6.5 (Tree Star).

### Immunofluorescence microscopy

Permanox chamber slides (Nunc Labtech) were coated with 0.1 mg/ml fibrinogen with or without 25 μg/ml of DQ collagen for 30 minutes. For experiments involving NETs, 10 Ü/ml DNase (Fermentas) was added for 20 minutes at room temperature at the end of the experiment. Samples were then fixed with 4% paraformaldehyde for 30 minutes and permeabilized with 0.5% saponin for 10 minutes. Cells were washed before blocking with 10% human AB serum with 2.5% BSA and 0.05% saponin. Primary antibodies were added overnight. Chamber slides were washed prior to the addition of secondary antibodies. The chambers were subsequently removed from the slide, and Fluoroshield Mouting medium with DAPI (Abcam) was added. Images were captured using Leica confocal microscope (Leica TCS SP5) and processed using Leica LAS AF Lite 2.6.0 (Leica Microsystems, Germany) and Image J 1.43U (NIH, USA).

### Immunohistochemistry

Five non immunosuppressed patients with biopsy proven pulmonary *M*.*tb* infection were analysed. The positive controls colon tumours (AMPK) from 10 patients and inflammed appendix (MMP-8). Negative controls were performed using the appropriate isotype control antibodies. Sections were immunostained for MMP-8 and phospho-AMPK alpha 1 and 2 (T172); neutrophil elastase with epitope retrieval performed by enzyme digestion using Bond Enzyme Pretreatment Kit. All antibodies were incubated for 15 minutes at room temperature. All immunohistochemistry was performed using the Leica Bond-III automated platform and associated ancillary reagents (all Leica Biosystems). The antibodies were detected using the Bond Polymer Refine Detection System and Bond DAB Enhancer according the manufacturer’s instructions.

### Statistical analyses

Data were analyzed using GraphPad Prism (version 5.04, GraphPad Software). Data are expressed as mean ± s.d. unless stated otherwise. All experiments are performed in biological triplicates on at least 2 separate occasions. Multiple intervention experiments are compared with one-way ANOVA followed by Tukey’s post-test correction, while continuous variables between 2 sets of data are assessed using two-tailed Mann-Whitney-U test. Spearman’s rank correlation tests are used for correlation analyses. *P* values of less than 0.05 are taken as statistically significant.

## Supporting Information

S1 Table Demographic data of healthy controls and TB patients. (DOCX)

S1 Fig (A) TIMP-1 secretion is not increased with *M*.*tb* multiplicity of infection (MOI) at 4 hours. (B) TIMP-2 concentrations increase in a dose-dependent manner with *M*.*tb* MOI at 4 hours. (C) NF-kB inhibition suppresses neutrophil MMP-8 secretion driven by *M*.*tb* infection. Neutrophils were preincubated with p65 unit inhibitor Helenalin for 30 minutes and stimulated with *M*.*tb* MOI of 10 for 4 hours. (D and E) NF-kB inhibition suppresses neutrophil MMP-8 secretion driven by *M*.*tb* infection. Neutrophils were pre-incubated with caffeic acid phenethyl ester (CAPE) or SN50 for 30 minutes and stimulated with *M*.*tb* MOI of 10 for 4 hours. Bars represent mean ± s.d. of experiments performed in biological triplicates and is representative of at least 2 experiments. Analysis done by one-way ANOVA. ** *P*<0.01, *** *P*<0.001.
(TIF)

S2 Fig NF-kB inhibition does not affect cell viability. Neutrophil viability of conditions for Fig 1D and 1E by FACS staining with Annexin V and live/dead dye. 50,000 events were gated. FACS plots representative of 2 donors.
(TIF)

S3 Fig Necrotic centre of granuloma is positive for MMP-8. Biopsy proven *M*.*tb* infected human lung specimens were stained for MMP-8 and matched isotype control antibody.
(TIF)

S4 Fig *M*.*tb* induces NETs. (A) Neutrophils were stimulated with *M*.*tb* MOI of 10 or 20nM PMA for 4 hours and NETs quantified using Picogreen QuantIT. All bars represent mean ± s.d. of experiments done in biological triplicates and are representative of a minimum of 2 independent experiments. (B and C) *M*.*tb* induced neutrophil extracellular traps are associated with MMP-8. Neutrophils were stimulated with PMA or infected with *M*.*tb* MOI 10 for 4 hours. DNAse was added into selected conditions. (D) Citrulline H3 is not associated with dead neutrophils. Neutrophils were either stimulated with PMA or lysed with Triton-X. 10μg of protein from cell-free supernatant were acetone precipitated and immunoblotted.
(TIF)

S5 Fig Gelatinase degradation of respiratory secretions from healthy controls and TB patients. (**A**) TB patients have increased gelatinase activity in their induced sputum samples. (n = 11 both groups). ****P<0.0001. (**B**) Anti-MMP-9 neutralizing antibody at final concentration of 10 μg/ml does not decrease gelatinase activity in the induced sputum of TB patients (n = 11).
(TIF)

S6 Fig Densitometric analysis from human protein kinase phosphoarray. Neutrophils were stimulated with CoMCont or CoMTB for 30 minutes. (A) Components of the MAP-kinase pathway. (B) Components of the STAT pathway. (C) Components of other signalling pathways. Protein kinase dots were normalized to control dots on each membrane. Bars represent mean from arrays of 4 human donors.
(TIF)

S7 Fig Neutrophil MMP secretion is independent of Akt/PI3 kinase and mTOR/p70S6K pathway. (A, D) Neutrophils were stimulated with CoMCont or CoMTB and lysed at specified time points. (B, C and E) Neutrophils were pre-incubated with Akt-inhibitor, LY 294002 or rapamycin prior to stimulation with CoMTB. P = NS. Bars represent mean ± s.d. of an experiment done in biological triplicates and is representative of a minimum of 2 independent experiments.
(TIF)
