# Supplementary material for: Neutrophil-Derived MMP-8 Drives AMPK-Dependent Matrix Destruction in Human Pulmonary Tuberculosis
Source: PLoS Pathog. 2015 May 21;11(5):e1004917. doi: 10.1371/journal.ppat.1004917 (PMC4440706; doi:10.1371/journal.ppat.1004917)
Supplement: S1 Table — (DOCX) [file ppat.1004917.s001.docx]

| **Variable ^a^** | **Control** | **TB** | ***P* value ^b^** |
| --- | --- | --- | --- |
| Number | 57 | 51 |  |
| Sex (M:F) n | 11 : 46 | 32 : 19 | <0.0001 |
| Age (years) | 28 (18-62) | 25 (18-72) | 0.1571 |
| BMI | 25.1 (18.5-37.5) | 20.9 (11.8-27.9) | <0.0001 |
| Pulse (per min) | 76 (61-104) | 96 (64-139) | <0.0001 |

a. Values are median (Interquartile range)

b. Comparison of continuous variables using Mann-Whitney U and categorical variables using Fisher’s exact.

**Table S1.** Demographic data of healthy controls and TB patients.
