# Supplementary figures and images for: Neutrophil-Derived MMP-8 Drives AMPK-Dependent Matrix Destruction in Human Pulmonary Tuberculosis
Source: PLoS Pathog. 2015 May 21;11(5):e1004917. doi: 10.1371/journal.ppat.1004917 (PMC4440706; doi:10.1371/journal.ppat.1004917)

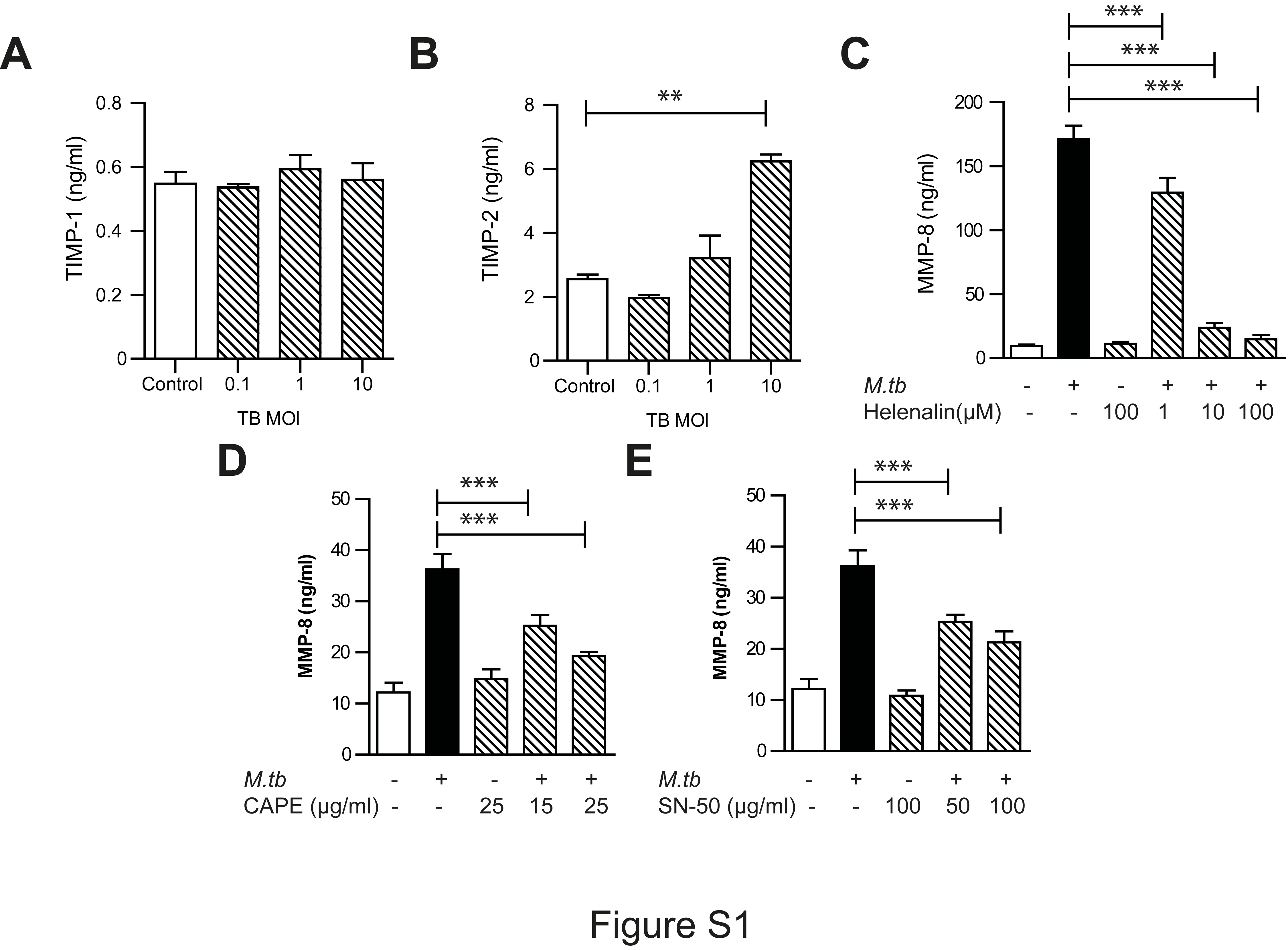

Supplement: S1 Fig — (B) TIMP-2 concentrations increase in a dose-dependent manner with M.tb MOI at 4 hours. (C) NF-kB inhibition suppresses neutrophil MMP-8 secretion driven by M.tb infection. Neutrophils were preincubated with p65 unit inhibitor Helenalin for 30 minutes and stimulated with M.tb MOI of 10 for 4 hours. (D and E) NF-kB inhibition suppresses neutrophil MMP-8 secretion driven by M.tb infection. Neutrophils were pre-incubated with caffeic acid phenethyl ester (CAPE) or SN50 for 30 minutes and stimulated with M.tb MOI of 10 for 4 hours. Bars represent mean ± s.d. of experiments performed in biological triplicates and is representative of at least 2 experiments. Analysis done by one-way ANOVA. ** P<0.01, *** P<0.001. (TIF) [file ppat.1004917.s002.tif]

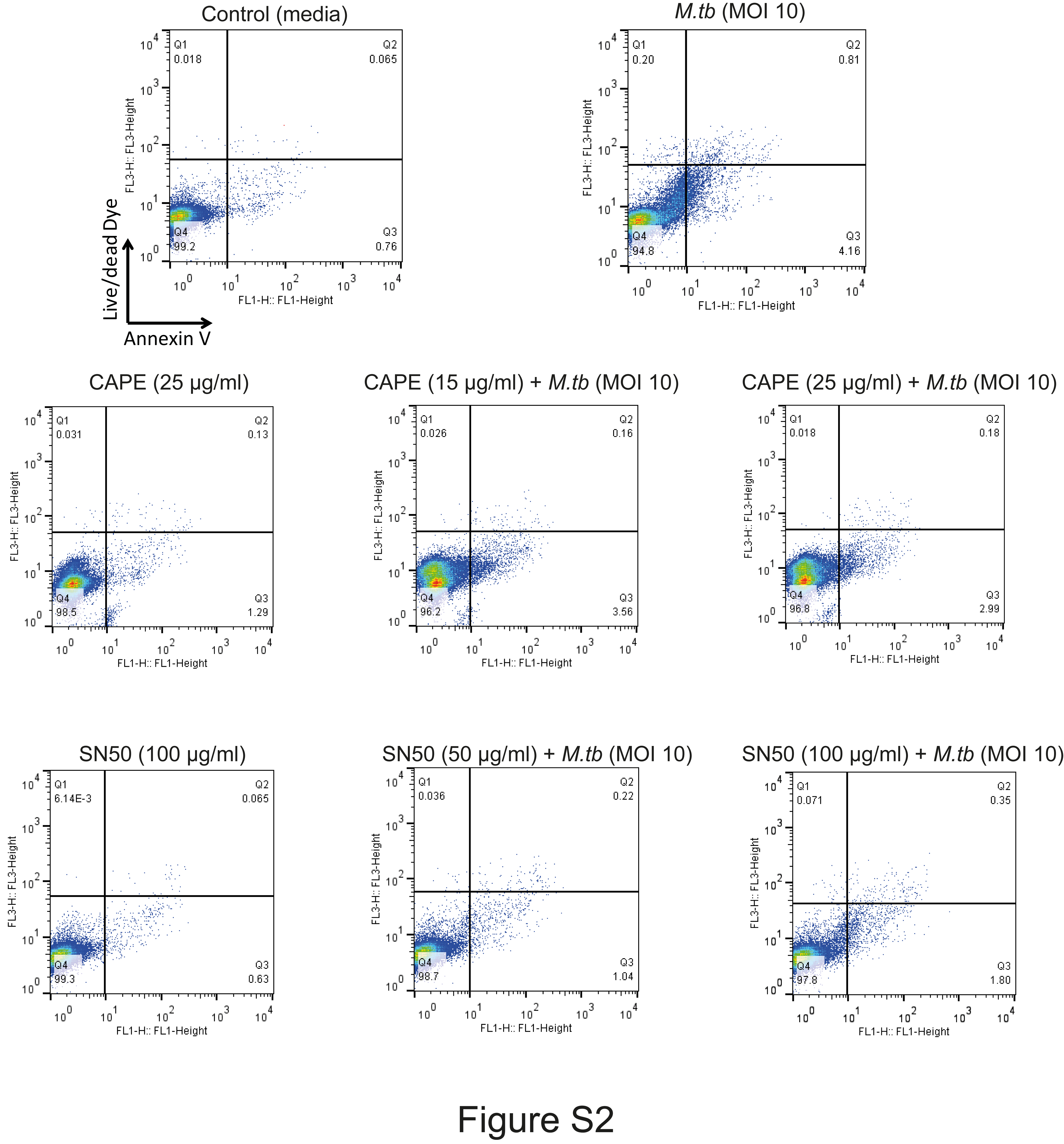

Supplement: S2 Fig — Neutrophil viability of conditions for Fig 1D and 1E by FACS staining with Annexin V and live/dead dye. 50,000 events were gated. FACS plots representative of 2 donors. (TIF) [file ppat.1004917.s003.tif]

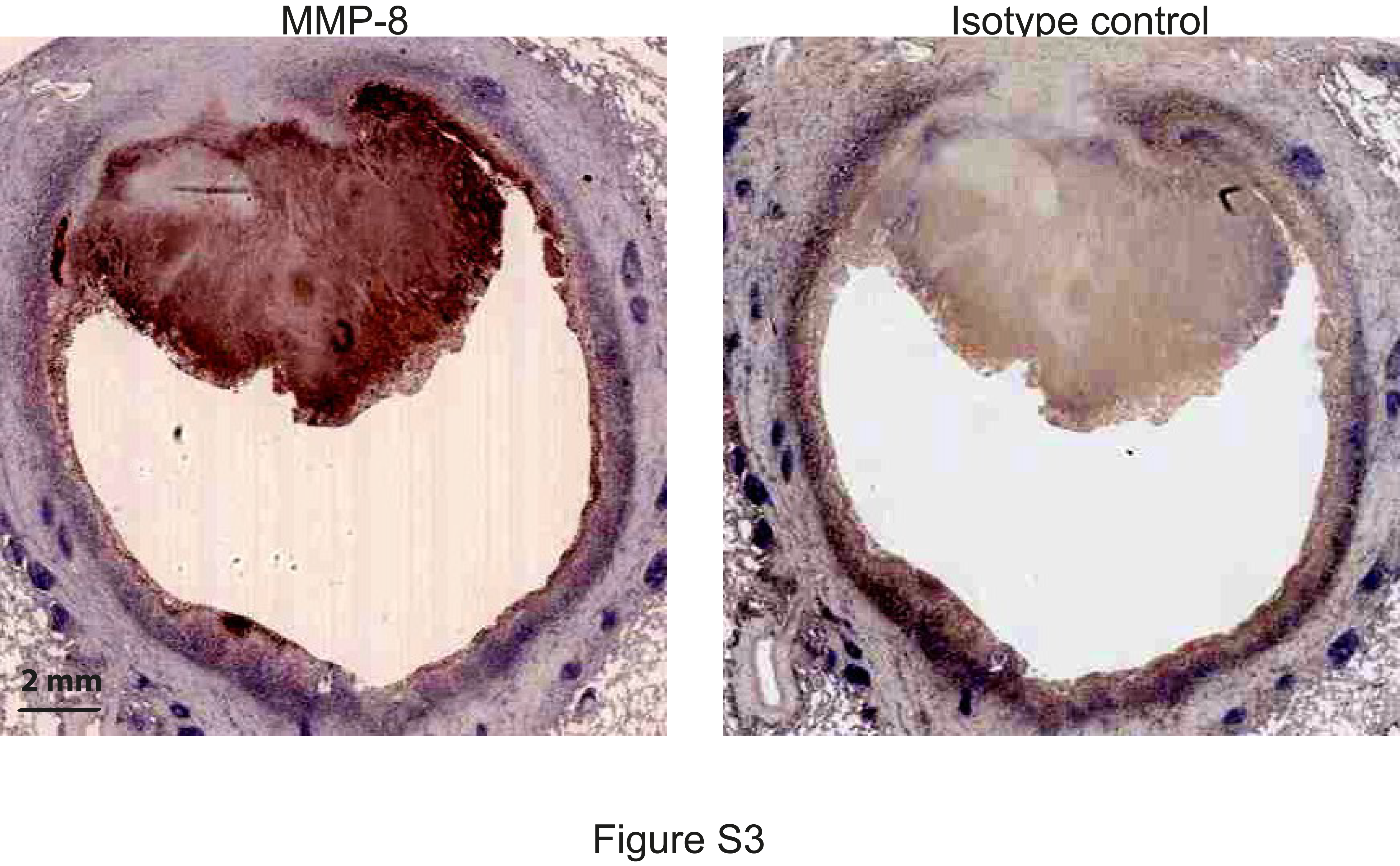

Supplement: S3 Fig — Biopsy proven M.tb infected human lung specimens were stained for MMP-8 and matched isotype control antibody. (TIF) [file ppat.1004917.s004.tif]

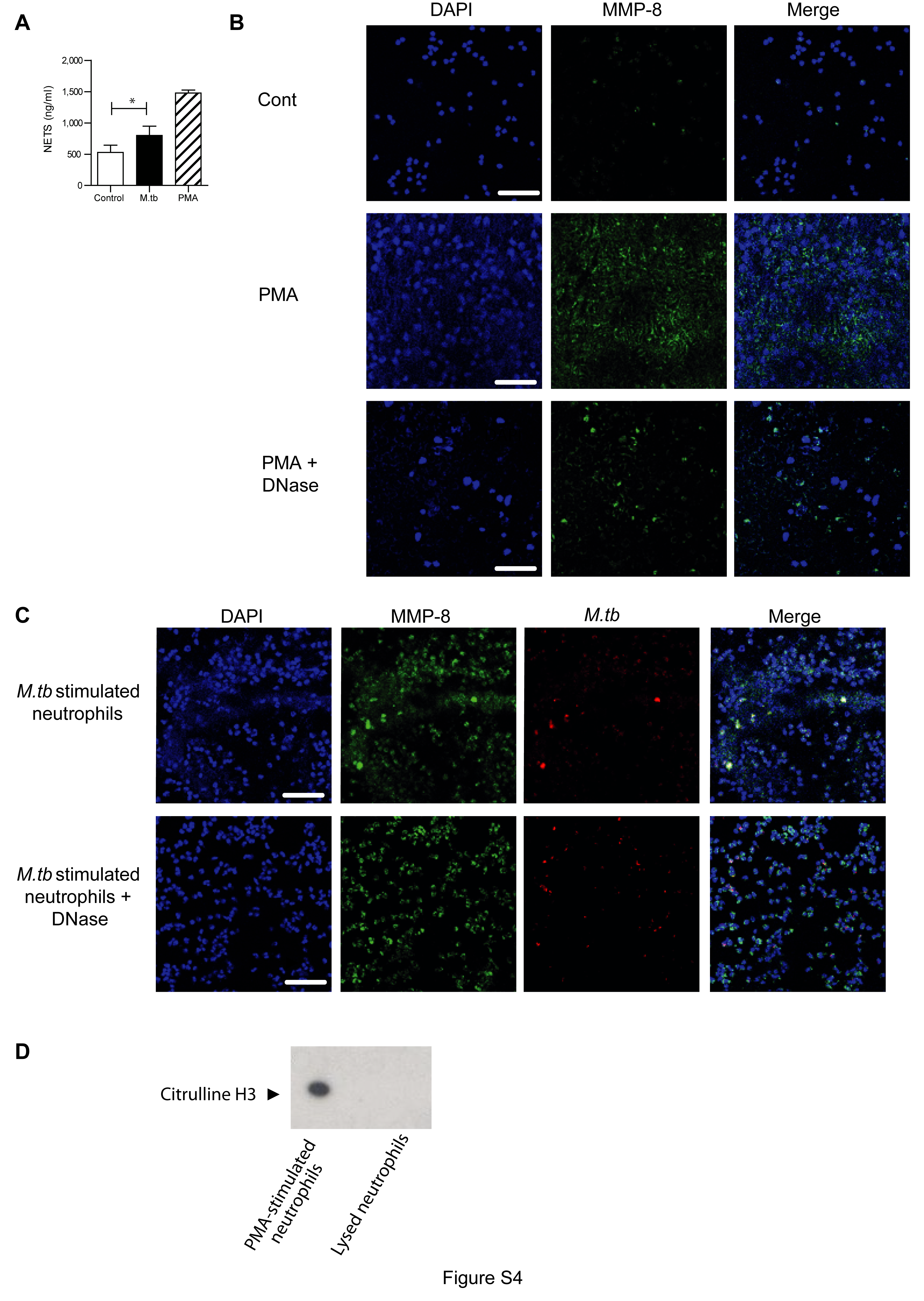

Supplement: S4 Fig — (A) Neutrophils were stimulated with M.tb MOI of 10 or 20nM PMA for 4 hours and NETs quantified using Picogreen QuantIT. All bars represent mean ± s.d. of experiments done in biological triplicates and are representative of a minimum of 2 independent experiments. (B and C) M.tb induced neutrophil extracellular traps are associated with MMP-8. Neutrophils were stimulated with PMA or infected with M.tb MOI 10 for 4 hours. DNAse was added into selected conditions. (D) Citrulline H3 is not associated with dead neutrophils. Neutrophils were either stimulated with PMA or lysed with Triton-X. 10μg of protein from cell-free supernatant were acetone precipitated and immunoblotted. (TIF) [file ppat.1004917.s005.tif]

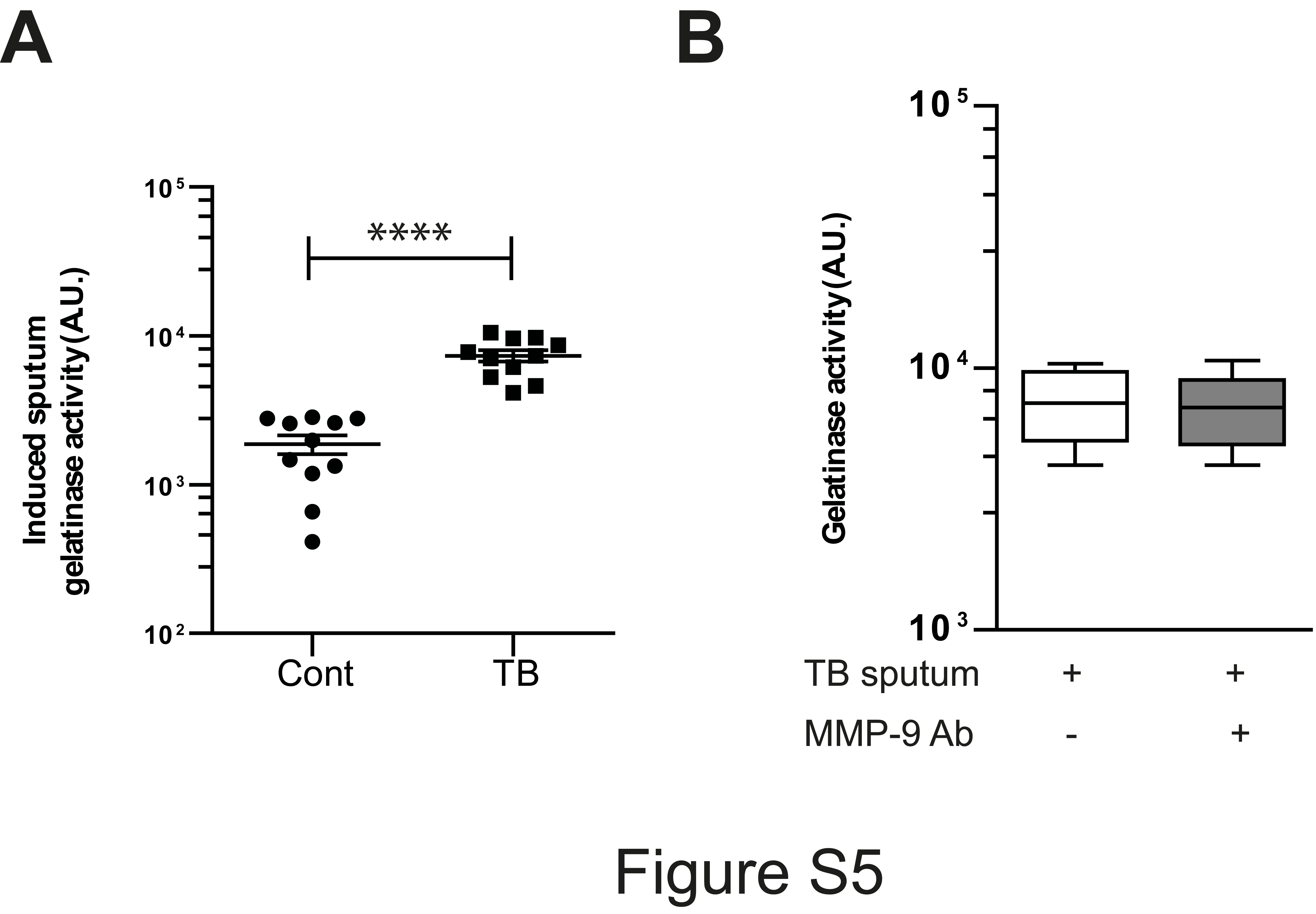

Supplement: S5 Fig — (A) TB patients have increased gelatinase activity in their induced sputum samples. (n = 11 both groups). ****P<0.0001. (B) Anti-MMP-9 neutralizing antibody at final concentration of 10 μg/ml does not decrease gelatinase activity in the induced sputum of TB patients (n = 11). (TIF) [file ppat.1004917.s006.tif]

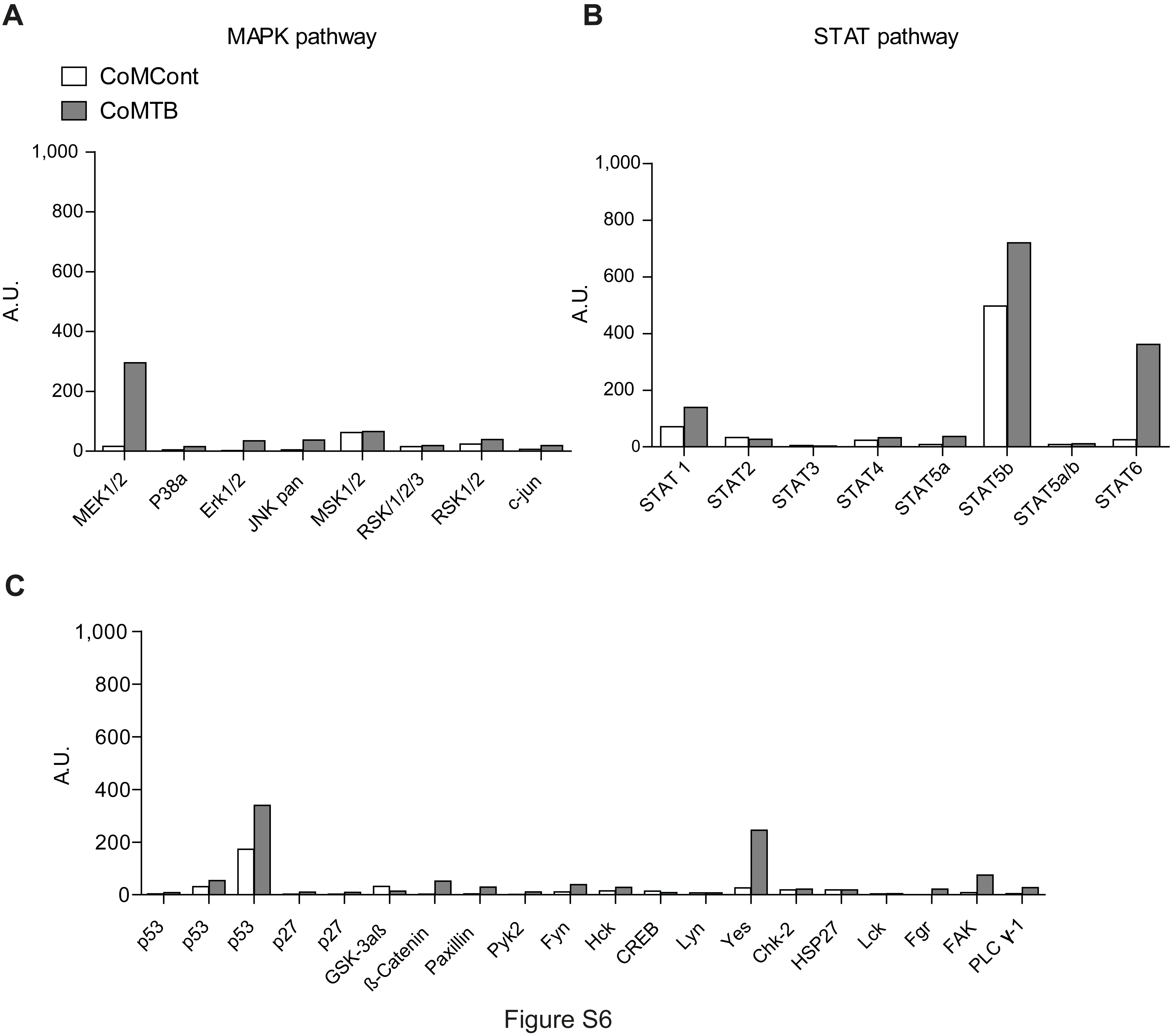

Supplement: S6 Fig — Neutrophils were stimulated with CoMCont or CoMTB for 30 minutes. (A) Components of the MAP-kinase pathway. (B) Components of the STAT pathway. (C) Components of other signalling pathways. Protein kinase dots were normalized to control dots on each membrane. Bars represent mean from arrays of 4 human donors. (TIF) [file ppat.1004917.s007.tif]

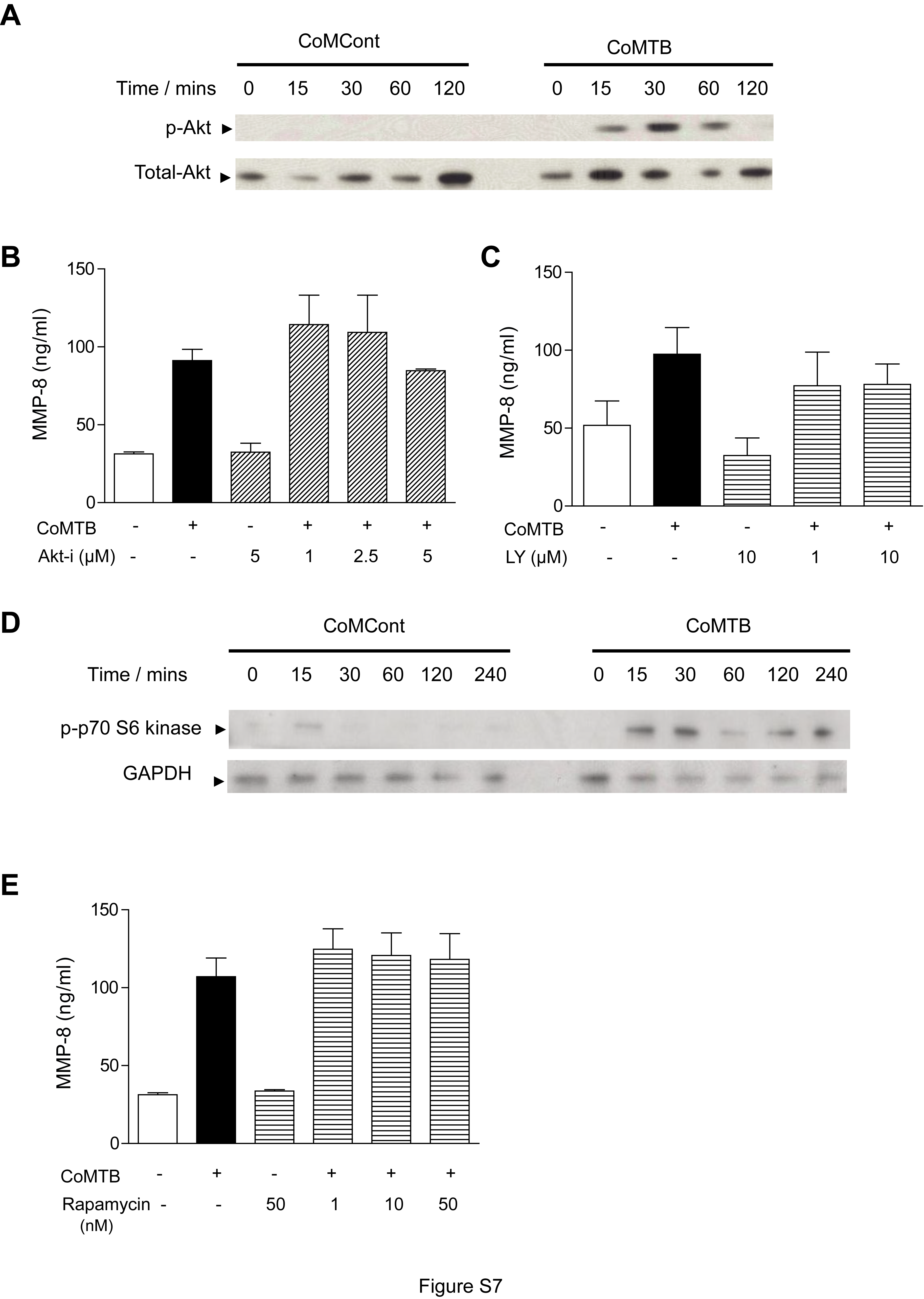

Supplement: S7 Fig — (A, D) Neutrophils were stimulated with CoMCont or CoMTB and lysed at specified time points. (B, C and E) Neutrophils were pre-incubated with Akt-inhibitor, LY 294002 or rapamycin prior to stimulation with CoMTB. P = NS. Bars represent mean ± s.d. of an experiment done in biological triplicates and is representative of a minimum of 2 independent experiments. (TIF) [file ppat.1004917.s008.tif]
